# The impact of sarcopenia on esophagectomy for cancer: a systematic review and meta-analysis

**DOI:** 10.1186/s12893-023-02149-6

**Published:** 2023-08-17

**Authors:** Amanda Park, Marina Feliciano Orlandini, Daniel José Szor, Ulysses Ribeiro Junior, Francisco Tustumi

**Affiliations:** 1 Department of Gastroenterology, Universidade de São Paulo (USP), São Paulo, São Paulo Brazil; 2 Centre for Evidence-Based Medicine, Centro Universitário Lusíada (UNILUS), Santos, Brazil

**Keywords:** Sarcopenia, Esophageal neoplasms, Esophagectomy, Systematic review, Meta-analysis

## Abstract

**Background:**

Esophagectomy is the gold-standard treatment for locally advanced esophageal cancer but has high morbimortality rates. Sarcopenia is a common comorbidity in cancer patients. The exact burden of sarcopenia in esophagectomy outcomes remains unclear. Therefore, this systematic review and meta-analysis were performed to establish the impact of sarcopenia on postoperative outcomes of esophagectomy for cancer.

**Methods:**

We performed a systematic review and meta-analysis comparing sarcopenic with non-sarcopenic patients before esophagectomy for cancer (Registration number: CRD42021270332). An electronic search was conducted on Embase, PubMed, Cochrane, and LILACS, alongside a manual search of the references. The inclusion criteria were cohorts, case series, and clinical trials; adult patients; studies evaluating patients with sarcopenia undergoing esophagectomy or gastroesophagectomy for cancer; and studies that analyze relevant outcomes. The exclusion criteria were letters, editorials, congress abstracts, case reports, reviews, cross-sectional studies, patients undergoing surgery for benign conditions, and animal studies. The meta-analysis was synthesized with forest plots.

**Results:**

The meta-analysis included 40 studies. Sarcopenia was significantly associated with increased postoperative complications (RD: 0.08; 95% CI: 0.02 to 0.14), severe complications (RD: 0.11; 95% CI: 0.04 to 0.19), and pneumonia (RD: 0.13; 95% CI: 0.09 to 0.18). Patients with sarcopenia had a lower probability of survival at a 3-year follow-up (RD: -0.16; 95% CI: -0.23 to -0.10).

**Conclusion:**

Preoperative sarcopenia imposes a higher risk for overall complications and severe complications. Besides, patients with sarcopenia had a lower chance of long-term survival.

**Supplementary Information:**

The online version contains supplementary material available at 10.1186/s12893-023-02149-6.

## Background

Esophagectomy is a major surgical procedure with an inherently high risk for postoperative complications [1]. The main complications are anastomotic leak, infection, paralysis of the vocal cords, pulmonary-related complications, and others [2, 3]. The postoperative mortality risk is around 5% [4]. Consequently, a rigorous preoperative risk surgical assessment is necessary to improve postoperative outcomes. In this setting, eligibility for the surgery depends on the patient’s general conditions, including caloric-protein nutritional status [5, 6].

Patients with esophageal cancer often present a malnutrition status. Esophageal cancer leads to obstructive symptoms, as the tumor mass prevents food passage and thus makes it impossible for the patient to intake the necessary calories and nutrients [7]. In addition, the metabolic and physical effects of cancer, with a chronic inflammatory state and excessive catabolism, as well as the side effects of anti-cancer treatments, contribute to cachexia and weight loss [8, 9].

Sarcopenia is a syndrome characterized by loss of strength and skeletal muscle mass [10]. The prevalence of preoperative sarcopenia in patients with esophageal cancer ranges from 14.4 to 80% [2]. The calculation of skeletal muscle mass (SMM), based on the skeletal muscle index (SMI) obtained by computed tomography of the transverse muscle mass at the level of the lumbar vertebras, is the gold standard test to diagnose sarcopenia [11]. Computed tomography is routinely ordered as a preoperative exam for esophageal cancer patients, and consequently, SMM is a promptly accessible and cheap test to investigate sarcopenia [11].

Sarcopenia is related to worse postoperative outcomes due to the increased risk of infection, physical disability, and deficit of tissue regeneration [2, 10]. Consequently, sarcopenia may pose a high risk for patients undergoing esophagectomy [10].

This systematic review and meta-analysis aim to increase the level of evidence with a quantitative synthesis of results that analyze the impact of sarcopenia on postoperative outcomes of patients with esophageal cancer submitted to curative resection.

## Methods

The systematic review and meta-analysis was reported and conducted in accordance with the PRISMA (Preferred Reporting Items for Systematic Review and Meta-Analysis) statement [12]. The study protocol was registered on PROSPERO (International Prospective Register of Systematic Reviews) [13] under the registration number CRD42021270332.

### Eligibility criteria

The inclusion criteria are cohort studies, case series, and clinical trials; adult patients (> 18 years old); studies evaluating patients with sarcopenia undergoing esophagectomy or gastroesophagectomy for cancer; and studies that analyze relevant outcomes.

The exclusion criteria are letters, editorials, congress abstracts, case reports, reviews, cross-sectional studies, patients undergoing surgery for reasons other than esophageal cancer, and animal studies.

### Information sources and search strategy

An online search was conducted in parallel and independently by two reviewers through PubMed, Embase, Cochrane Library Central, and Lilacs (BVS), alongside a manual search of references from all included studies, previous systematic reviews and meta-analyses. The search strategy was developed from the databases’ inception to December 2022 based on a combination of MeSH terms and keywords on Medline and Embase ((esophagectom* OR esophageal resection OR esophag* excision OR esophagus resection OR esophag* removal OR oesophago-gastrectomy OR oesophagectom*) AND (sarcopen* OR muscle loss OR muscle dystrophy OR muscle atrophy OR muscle atrophies OR muscle weakness OR muscle wasting OR muscle degeneration OR muscular loss OR muscular degeneration OR muscular atrophies OR muscular dystrophy OR cachexia OR cachectic) ; Lilacs ((sarcopen* OR muscle loss OR perda muscular OR muscle atroph* OR atrofia muscular OR cachexia) AND (esophagectom* OR oesophago-gastrectomy OR esophageal resection OR oesophagectomy OR (esophageal AND surgical resection)) AND (esophagus tumor OR esophagus cancer OR câncer de esôfago OR malign esophagus)); Cochrane ((esophagectom* OR esophageal resection OR esophageal excision OR esophagus excision OR esophagus removal OR oesophago-gastrectomy) AND (sarcopen* OR muscle loss OR muscle weakness OR muscle wasting OR muscular loss)).

### Study selection

Two reviewers conducted the study selection in parallel and independently. In case of conflict concerning the inclusion of a study, a third more experienced reviewer solved it after a group discussion where both parties were taken into consideration. The study selection was initially by title evaluation, abstract, and later by full-text analysis, following the predefined eligibility criteria. No restrictions were applied on either language or period of publication. No filters were used for selection.

### Data extraction

The baseline characteristics of the included studies were extracted, such as mean age, sex, esophageal cancer type, clinical staging, neoadjuvant therapy, type of esophagectomy, and the outcomes-related variables, such as postoperative mortality, postoperative complications, anastomotic leak, length of hospital stay, and length of ICU stay.

### Statistical analysis and data synthesis

Data were manually extracted independently by two reviewers and then meta-analyzed using the Software STATA 16.0 (StataCorp LLC). The summary results were expressed as risk difference (RD) for categorical variables and mean differences (MD) for continuous variables. A 95% confidence interval was applied. Statistical heterogeneity was evaluated using the I^2^ test A random effect model was applied to weigh the statistical and clinical heterogeneity. The meta-analysis was synthesized with forest plots.

In addition, a subset of studies that assessed sarcopenia with Skeletal Muscle Mass Index (SMI) was performed to investigate the robustness of the meta-analysis. Both fixed and random effect models were applied for this subset of studies as sensitivity analyses.

### Risk of bias assessment

All eligible studies considered went through the risk of bias assessment by the Newcastle Ottawa scale [14], a tool typically used for assessing the quality of non-randomized studies. Risk of bias and quality assessment was conducted by two independent reviewers. If there is any disagreement, a third reviewer made the decision after a group discussion where both parties were taken into consideration.

### Outcomes

The following outcomes were analyzed: postoperative mortality, postoperative complications, anastomotic leak, length of hospital stay, and length of ICU stay.

## Results

### Study selection and characteristics

**Fig. 1:**
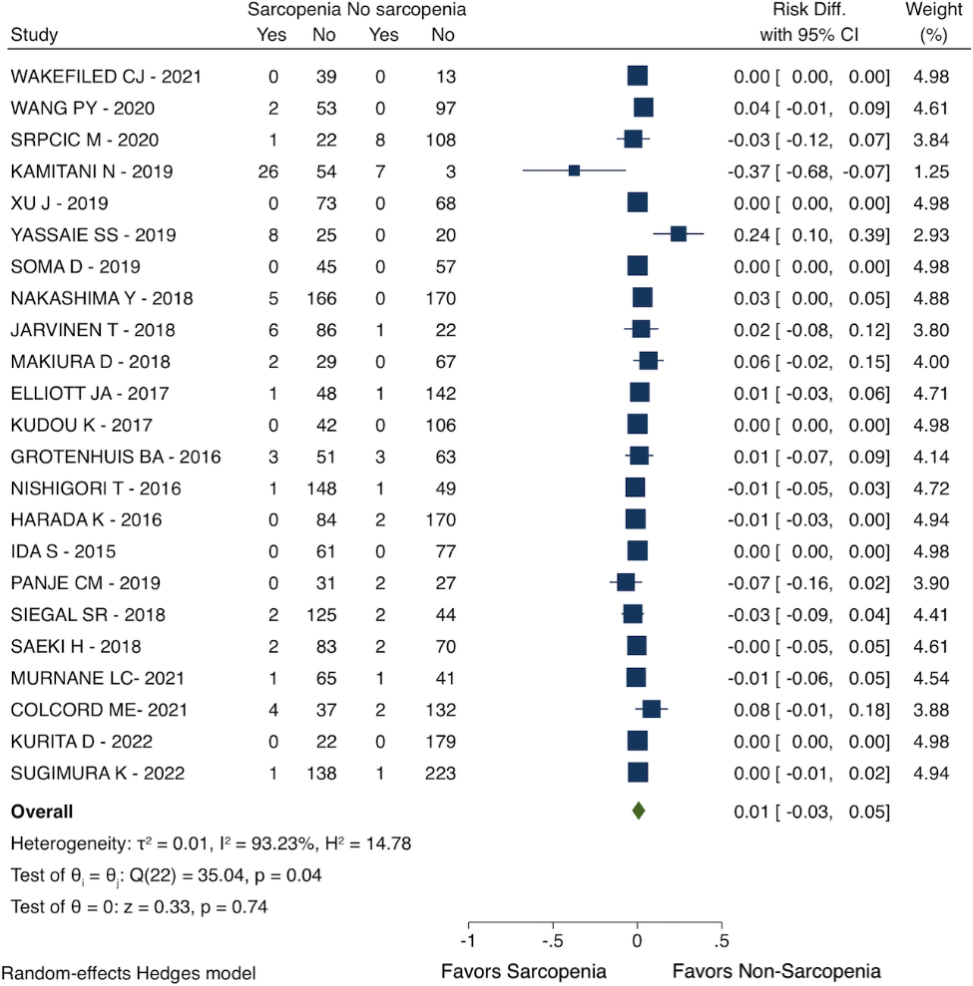
PRISMA 2020 Flowchart

As detailed in the selection flow diagram (Fig. 1), the initial search yielded 2804 results. After the removal of duplicate records and ineligible studies, 103 remained and were fully reviewed based. Of these, 40 were included [15–54], comprising 5669 patients from retrospective and prospective observational data.

The mean age across the studies was 65 years, with male predominance (82%). The baseline characteristics of the included studies are reported in Table 1.

**Table 1:** Baseline characteristics of the included studies. MIE: Minimally invasive esophagectomy; VATS: Video-assisted thoracoscopic surgery; SCC: Squamous cell carcinoma; CRT: Chemoradiotherapy; R: Retrospective; P: Prospective; NI: Not informed; SMM: Skeletal muscle mass; SMI: Skeletal muscle mass index; BMI: Body mass index; PMI: Psoas muscle index; MMI: Masseter mass index; BIA: Bioelectrical impedance analysis; HGS: Hand grip strength; CT: Computed tomography; TPA: Total psoas area

Author - year	N	Age (years)	Design	Male	Follow-up	MIE, hybrid, or VATS	SCC	Neoadj. CRT	Stage III/IV	Method	Sarcopenia definition	
											Men	Women
**NAMBARA M − 2021**	73	65.3	R	0.53	NI	0.88	NI	0.68	0.52	BIA	SMM < 90%	
**FEHRENBACH U − 2021**	85	64.3	R	0.88	24	0.91	0	0.21	0.67	CT L3	SMI ≤ 52.4 cm^2^/m^2^	SMI ≤ 38.5 cm^2^/m^2^
**UEMURA S − 2021**	121	65	R	0.83	36	0.99	NI	0.3	NI	CT L3	PMI < 6 cm^2^/m^2^, BMI < 18.5 kg/m^2^	
**WAKEFILED CJ − 2021**	52	65	R	0.87	24.9	0.23	0.17	NI	0.15	CT L3	SMI: < 43 cm^2^/m^2^ (BMI < 25), 53 cm^2^/m^2^ (BMI ≥ 25)	SMI < 41 cm^2^/m^2^
**MAYANAGI S − 2020**	187	62.8	R	0.8	0.5	0.87	0.90	0.43	0.36	CT L3	PMI < 6.36 cm^2^/m^2^	PMI < 3.92 cm^2^/m^2^
**WANG PY − 2020**	152	64.6	P	0.67	3	1	0.93	0.40	0.61	NI	Low appendicular skeletal muscle mass index < 7 kg/m^2^	Low appendicular skeletal muscle mass index < 5.7 kg/m^2^
**MAEDA N − 2020**	72	66	R	0.9	NI	0.69	1	0.01	0.5	CT L3	SMI < 52.4 cm^2^/m^2^	SMI < 38.5 cm^2^/m^2^
**SAKAI M − 2021**	89	64.1	R	0.87	60	NI	0.93	0	0.48	CT L3	SMI < 52.4 cm^2^/m^2^	SMI < 38.5 cm^2^/m^2^
**KAWAKITA Y − 2020**	113	64.2	R	0.85	3	1	1	1	NI	CT L3	PMI < 3.85 cm^2^/m^2^	PMI < 2.42 cm^2^/m^2^
**SRPCIC M − 2020**	139	63.9	P	0.84	18.1	0.37	0.46	0.53	0.44	CT L3	SMI < 43.1 cm^2^/m^2^	SMI < 32.7 cm^2^/m^2^
**MENEZES TM − 2020**	26	58	P	0.65	NI	0.69	NI	1	0.42	CT L3	MMI 43 cm^2^/m^2^ (BMI < 25), 53 cm^2^/m^2^ (BMI ≥ 25). PTA < 545 mm^2^/m^2^	MMI 41 cm^2^/m^2^. PTA < 285 mm^2^/m^2^
**ISHIDA T − 2019**	165	65.9	R	0.87	NI	0.25	NI	1	0.70	CT L3	PMI < 6.36 cm2/m^2^	PMI < 3.92 cm^2^/m^2^
**KAMITANI N − 2019**	90	66.2	R	0.86	20.8	NI	1	1	NI	CT L3	SMI < 52.4 cm^2^/m^2^	SMI < 38.5 cm^2^/m^2^
**OGUMA J − 2019**	194	64	R	0.88	38	0.27	1	0	NI	CT L3	SMI < 52.4 cm^2^/m^2^	SMI < 38.5 cm^2^/m^2^
**XU J − 2019**	141	59.6	R	0.79	12	1	1	0	NI	CT L3	SMI < 52.4 cm^2^/m^2^	SMI < 38.5 cm^2^/m^2^
**YASSAIE SS − 2019**	53	64.5	R	0.92	NI	0	1	1	0.49	CT L4	> 4% loss of TPA	
**SOMA D − 2019**	102	68	R	0.87	NI	0.63	1	0.24	0.58	CT L3	SMI < 43 cm^2^/m^2^ (BMI < 25 kg/m^2^), < 53 cm^2^/m^2^ (BMI > 25 kg/m^2^)	SMI < 41cm^2^/m^2^
**MATSUNAGA T − 2019**	163	65	R	0.78	27	NI	0.94	0.07	0.36	NI	SMM < lower limit of standard SMM (< 90% of the standard)	
**NAGATA K − 2018**	123	70	R	0.85	60	0.67	1	NI	0.39	CT L3	PMI < 4.24 cm^2^/m^2^	PMI < 2.50 cm^2^/m^2^
**NAKASHIMA Y − 2018**	341	NI	R	0.85	60	NI	0.95	0.52	0.47	CT L3	SMI < 47.24 cm^2^/m^2^	SMI < 36.92 cm^2^/m^2^
**JARVINEN T − 2018**	115	63	R	0.75	24	0.77	0.23	1	NI	CT L3	SMI < 52.4 cm^2^/m^2^	SMI < 38.5 cm^2^/m^2^
**MAKIURA D − 2018**	98	65.6	R	0.85	24	0.93	NI	0.72	0.49	NI	Low muscle mass (< 7.0 kg/m^2^) plus low muscle strength (< 26 kg) and/or low physical performance (< 0.8 m/s)	Low muscle mass (< 5.7 kg/m^2^) plus low muscle strength (< 18 kg) and/or low physical performance (< 0.8 m/s)
**ELLIOTT JA − 2017**	192	61.6	P	0.82	26	NI	0.20	1	NI	CT L3	SMI < 52.4 cm^2^/m^2^	SMI < 38.5 cm^2^/m^2^
**KUDOU K − 2017**	148	64.9	R	0.72	60	0.06	0	NI	NI	CT L3	SMI < 43 cm^2^/m^2^ (BMI < 25 kg/m^2^), < 53 cm^2^/m^2^ (BMI > 25 kg/m^2^)	SMI < 41 cm^2^/m^2^
**PAIREDER M − 2017**	130	62.8	R	0.82	21.2	NI	0.33	0.05	NI	CT L3	SMI ≤ 55 cm^2^/m^2^	SMI ≤ 39 cm^2^/m^2^
**GROTENHUIS BA − 2016**	120	61.8	R	0.74	20	0	0.26	1	NI	CT L3	SMI < 52.4 cm^2^/m^2^	SMI < 38.5 cm^2^/m^2^
**MAKIURA D − 2016**	104	65.4	R	0.85	NI	NI	0.94	0.78	0.44	BIA + HGS	Low muscle mass (< 7.0 kg/m^2^) plus low muscle strength (< 26 kg) and/or low physical performance (< 0.8 m/s)	Low muscle mass (< 5,7 kg/m^2^) plus low muscle strength (< 18 kg) and/or low physical performance (< 0.8 m/s)
**NISHIGORI T − 2016**	199	65.3	R	0.82	NI	0.94	1	0.58	0.34	CT L3	SMI < 52.4cm^2^/m^2^	SMI < 38.5 cm^2^/m^2^
**TAMANDL D − 2016**	200	64	R	0.75	35.1	NI	0.3	0.65	NI	CT L3	SMI ≤ 55cm^2^/m^2^	SMI ≤ 39 cm^2^/m^2^
**HARADA K − 2016**	256	NI	R	0.92	49.6	0.05	1	0.41	0.59	CT L3	SMI < 44.5 cm^2^/m^2^	SMI < 36.5 cm^2^/m^2^
**IDA S − 2015**	138	65.2	P	0.88	NI	NI	1	0.11	0.25	BIA	SMM < 90% of the standard	
**PANJE CM − 2019**	60	61	R	0.93	48	NI	0.18	1	0.72	CT L3	SMI < 43 cm^2^/m^2^ (BMI < 25 kg/m^2^), < 53 cm^2^/m^2^ (BMI > 25 kg/m^2^)	SMI < 41cm^2^/m^2^
**SIEGAL SR − 2018**	173	65.6	R	0.83	27.6	0.95	0.12	0.83	0.47	CT L3	SMI < 52.4 cm^2^/m^2^	SMI < 38.5 cm^2^/m^2^
**SAEKI H − 2018**	157	64	R	0.82	60	NI	1	0.53	0.76	CT L3	SMI < 52.4 cm^2^/m^2^	SMI < 38.5 cm^2^/m^2^
**MURNANE LC- 2021**	108	66	R	0.75	NI	NI	0.11	0.21	NI	CT L3	SMI < 52.4 cm^2^/m^2^	SMI < 38.5 cm^2^/m^2^
**COLCORD ME- 2021**	**175**	67	R	0.86	12	1	0.13	0.84	NI	Hand-grip	< 26 kg	< 16 kg
**KAMADA T − 2022**	70	68	R	0.93	36	1	0.88	0.06	0.4	Masseter	< 24.3 cm^2^	< 26.2 cm^2^
**WATANABE A − 2022**	135	67	R	0.67	NI	1	NI	0.69	0.43	CT L3	Low muscle mass < 7.0 kg/m^2^	Low muscle mass < 5.7 kg/m^2^
**KURITA D − 2022**	247	65	R	0.81	NI	1	0.66	0	0.51	Hand-grip	< 28 kg	< 18 kg
**SUGIMURA K − 2022**	363	70	R	0.8	33	NI	NI	0.16	0.35	NI	Low muscle mass < 7.0 kg/m^2^	Low muscle mass < 5.7 kg/m^2^

Quality assessment using the New-castle-Ottawa Scale demonstrated that all the included studies scored 5 or 6 points out of 9 (Supplementary File 1).

### Postoperative mortality

**Fig. 2:**
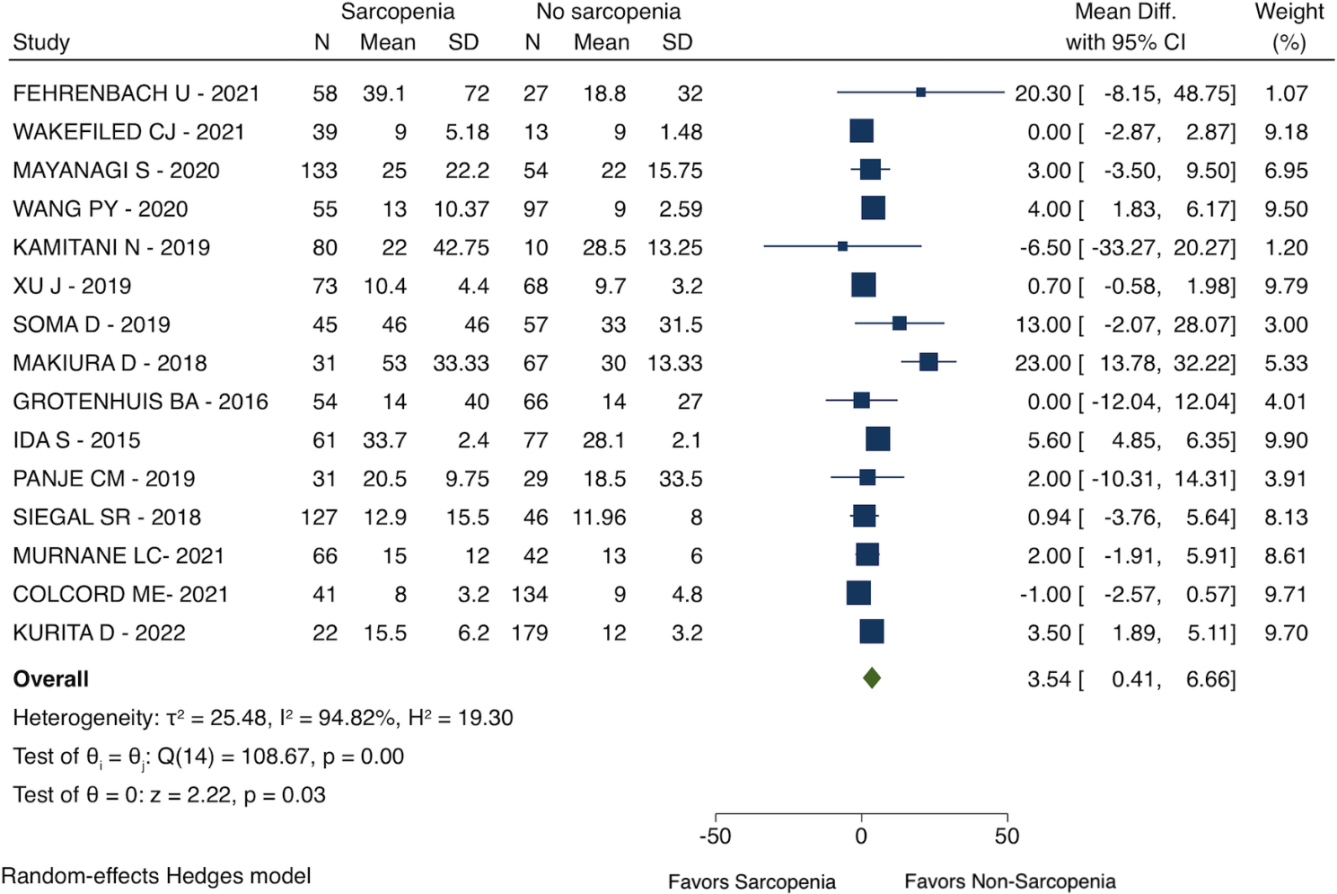
Postoperative mortality

Patients with sarcopenia had a similar all-cause mortality rate compared with non-sarcopenic patients after esophagectomy (RD: 0.01; 95% CI: -0.03 to 0.05; I^2^ = 93.23%; 23 studies with 3573 patients; see Fig. 2).

### Postoperative complications

**Fig. 3:**
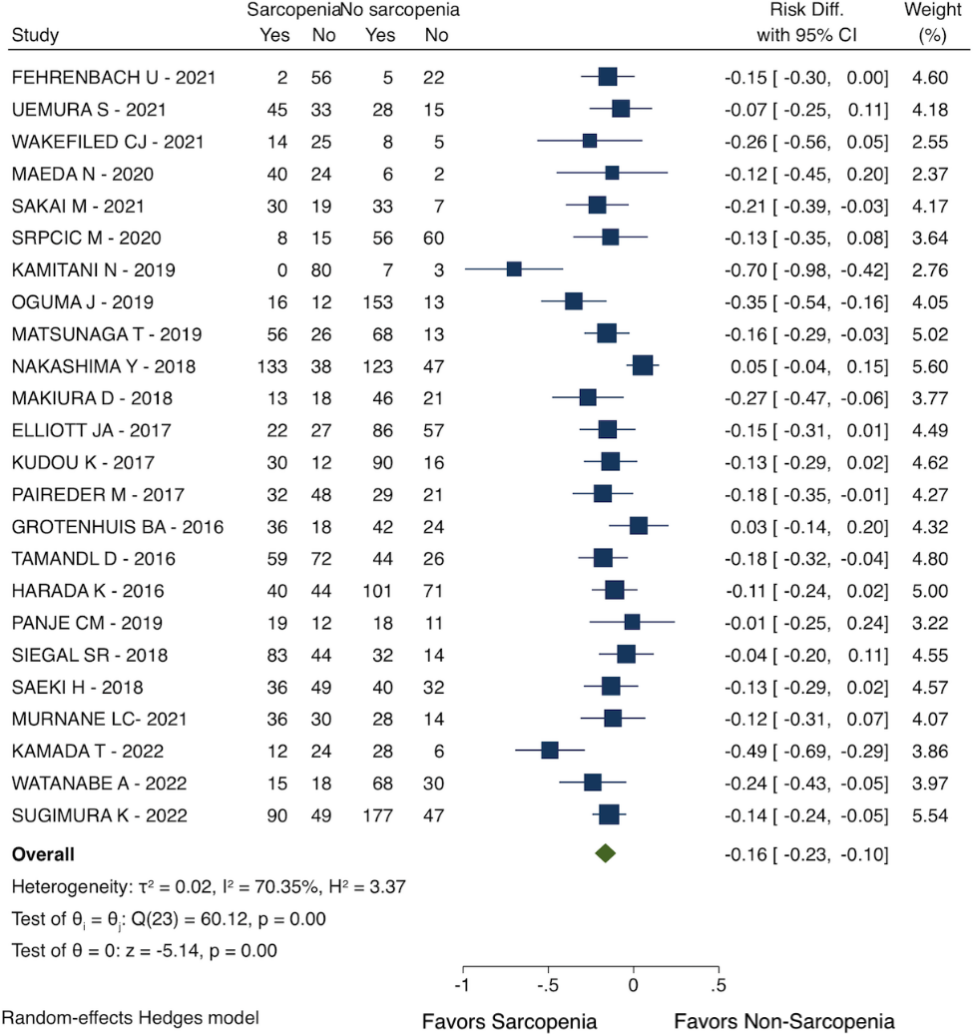
Postoperative complications. (**a**) Overall complications; (**b**) Severe complications (Clavien-Dindo > IIIa)

**Fig. 4:**
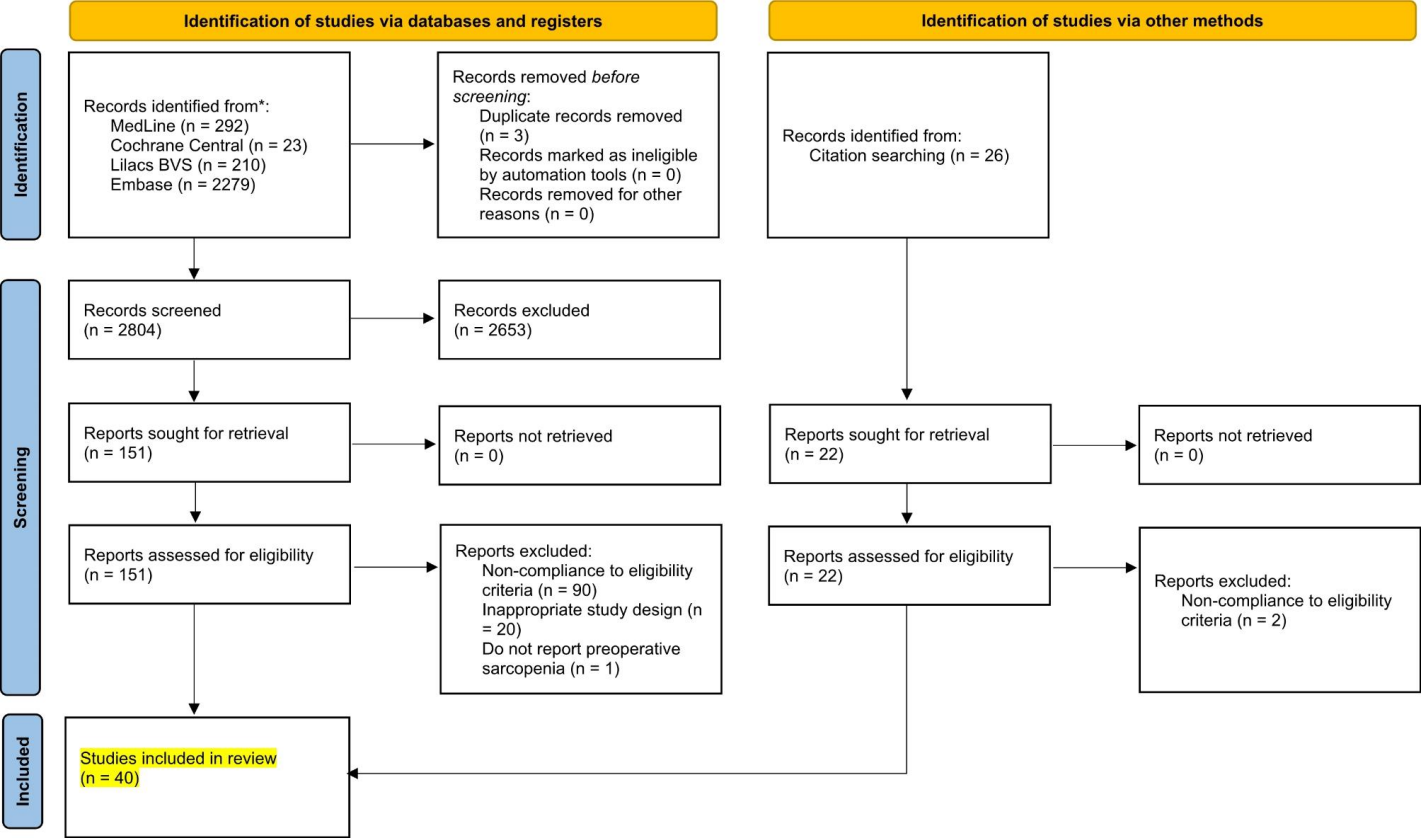
Postoperative complications. (**a**) Anastomotic leak; (**b**) Pneumonia

Sarcopenia before the esophageal surgery was related to an increased risk for overall complications (RD: 0.08; 95% CI: 0.02 to 0.14; I^2^ = 67.69%; 24 studies with 3767 patients; see Fig. 3a), and a higher risk for severe complications (Clavien-Dindo > IIIa) (RD: 0.11; 95% CI: 0.04 to 0.19; I^2^ = 68.90%; 10 studies with 1489 patients; see Fig. 3b). It was reported an increased risk for pneumonia (RD: 0.13; 95% CI: 0.09 to 0.18; I^2^ = 63.66%; 21 studies with 3062 patients; see Fig. 4b). However, the anastomotic leakage rate was similar between the two groups (RD 0.01; 95% CI: -0.01 to 0.02; I^2^ = 0,00%; 28 studies with 4316 patients; see Fig. 4a).

### Length of hospital stay

**Fig. 5:**
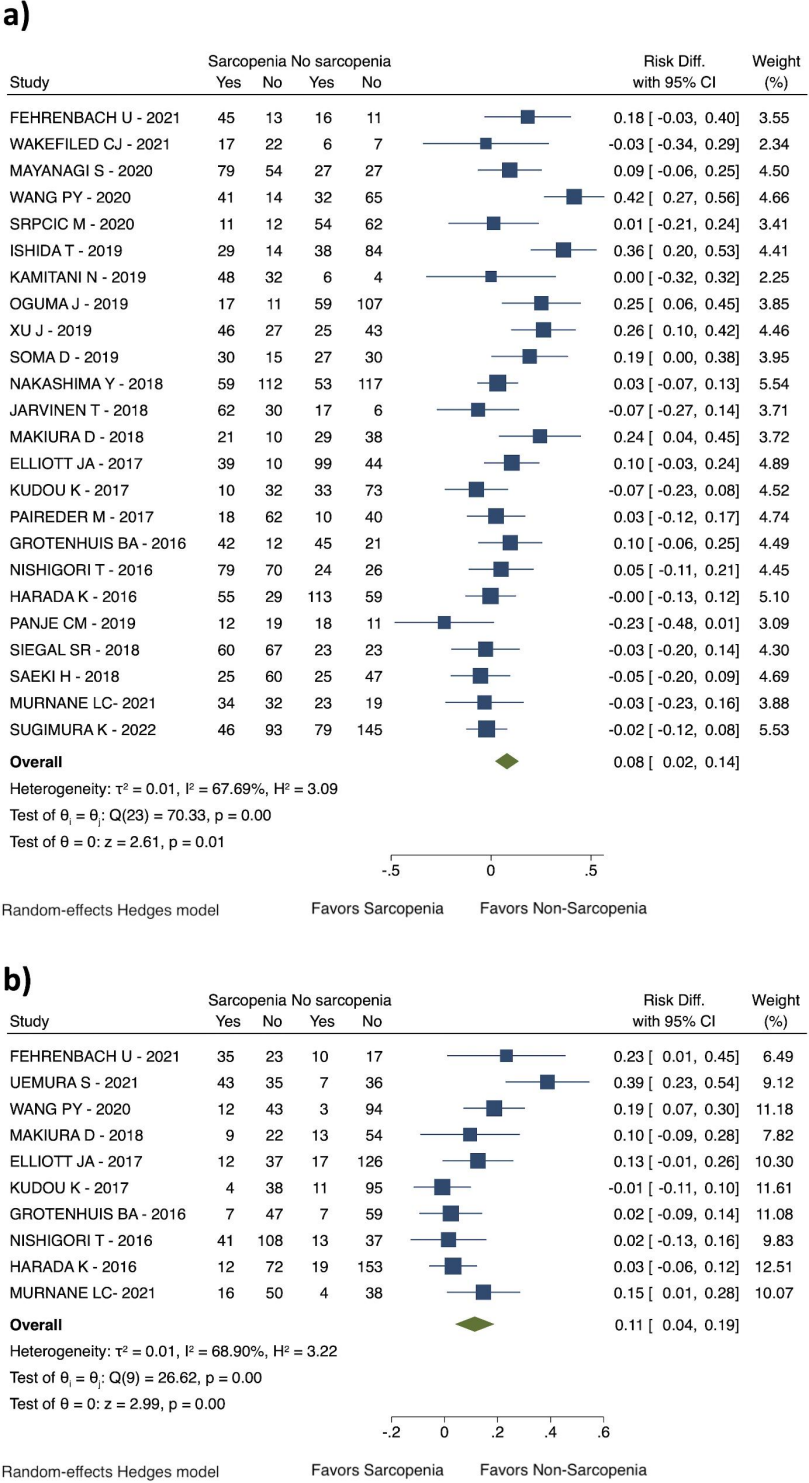
Length of hospital stay

Patients with sarcopenia had a longer length of hospital stay (MD: 3.54 days; 95% CI: 0.41 to 6.66; I^2^ = 94.82%; 15 studies with 1882 patients; see Fig. 5) than patients with no sarcopenia before esophagectomy.

### Overall survival

**Fig. 6:**
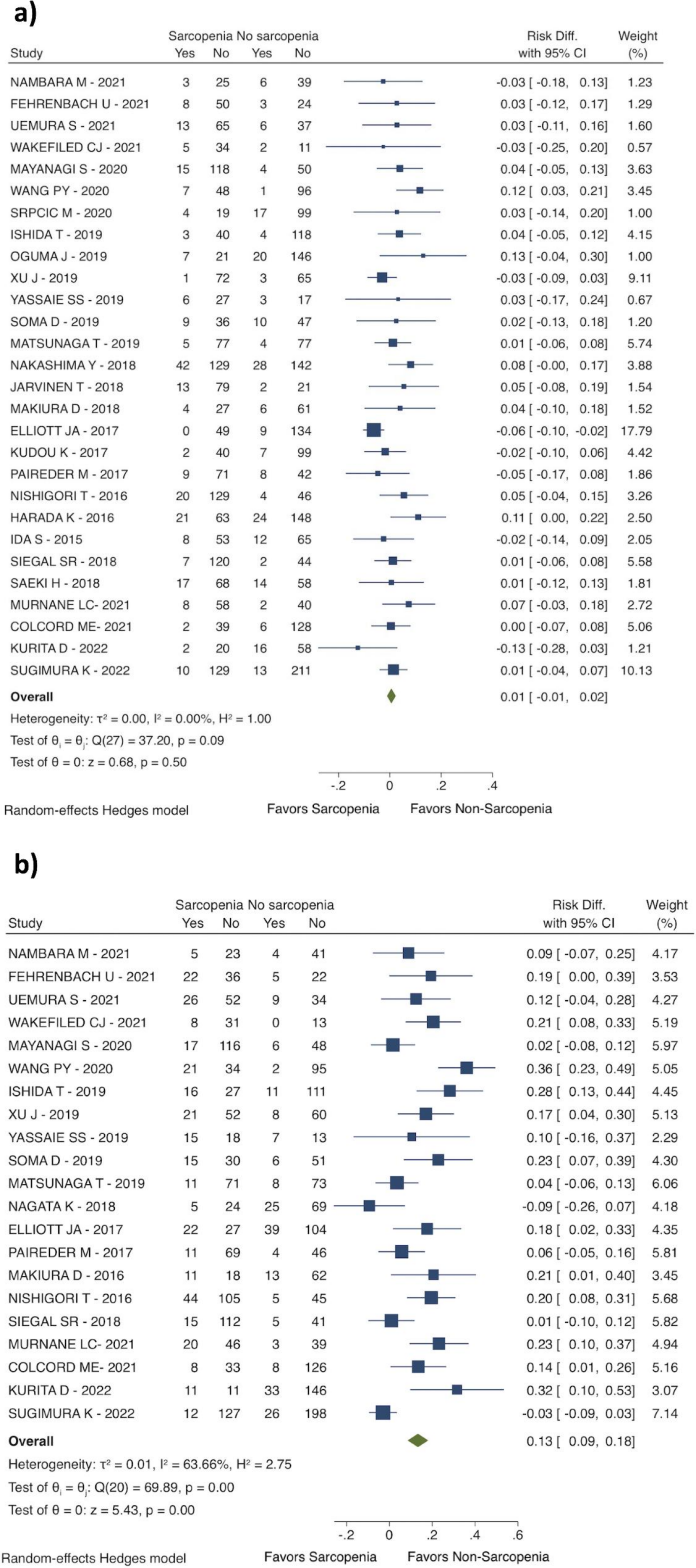
Overall survival (3-year follow-up)

Patients with sarcopenia had a lower probability of survival at 3-year follow-up (RD: -0.16; 95% CI: -0.23 to -0.10; I^2^ = 70.35%; 24 studies with 3504 patients, see Fig. 6).

### Sensitivity analysis

A subset analysis of studies that assessed sarcopenia using a cutoff for SMI ≤ 38.5 cm^2^/m^2^ in women and ≤ 52.4 cm^2^/m^2^ in men showed a reduction in I^2^ values. The direction and significance of the results were consistent for all endpoints except postoperative overall complications. The subgroup analyses found a significant impact of sarcopenia on overall complications, both in the fixed and random effect models. (Supplementary File 2).

## Discussion

In this systematic review and meta-analysis, we compared sarcopenic with non-sarcopenic patients who underwent esophagectomy for cancer. Preoperative sarcopenia was related to poor short- and long-term postoperative outcomes.

A variety of methods have been used to evaluate nutrition in esophageal cancer patients. Anthropometric measurements, blood indicators, energy expenditure, validated nutritional risk score, and patient-reported dietary history could be generally categorized among them [55, 56]. However, blood biomarkers of malnutrition may be affected by systemic therapies, and anthropometric measurements may fail in detecting early signs of muscle loss or in detecting malnutrition among patients with fluid disturbance, such as those with hypoalbuminemia [57, 58]. The current review focuses on the assessment of muscle mass.

The decrease in skeletal muscle mass, strength, and physical performance, known as sarcopenia, has been linked to several consequences in the human body [59, 60], making patients vulnerable to adverse outcomes. Muscle tissue is essential for protein storage, regulation of glucose metabolism, the balance of hormones, and the immunological system, aside from mobilization [61].

Our review showed that sarcopenia before oncological esophagectomy was linked to a higher risk for postoperative complications, mainly pneumonia. However, it not only negatively influenced esophageal cancer, but also the other types of cancer surgery [62, 63]. Weakening of the muscles responsible for changing the volume of the thoracic cavity during respiration may favor low thoracic expansibility during the postoperative period, which in turn leads to a higher risk for atelectasis, pleural effusion, and pneumonia [64]. Besides, loss of thoracic wall muscles may also contribute to extubation failure and prolonged mechanical ventilation [65]. The length of mechanical ventilation is directly related to the risk of ventilator-associated pneumonia. Chastre et al. [66] showed that the cumulative risk for pneumonia caused by *Acinetobacter spp*. in patients under mechanical ventilation is 3.4, 20, and 48% at 10, 20, and 30 days after the intubation, respectively.

Generalized sarcopenia of skeletal muscles also reflects in swallowing muscles. This condition is named sarcopenic dysphagia [67]. Loss of strength in the swallowing muscles may also contribute to aspiration pneumonia [68] and enhance perioperative malnutrition due to dysphagia, leading to a vicious cycle of sarcopenic dysphagia and malnutrition.

The limb and trunk skeletal muscle loss also impacts the patient’s capacity for early ambulation. The mobilization is inherently challenging in the postoperative course of an esophagectomy due to the restrictions imposed by thoracic drains, catheters, pumps, central lines, feeding tubes, and pain. Patients who delay mobilization have an increased incidence of pulmonary conditions, infectious complications, extended hospitalization, and a decreased home discharge rate [69, 70]. In addition, bed rest enhances muscle loss and sarcopenia [71], creating another vicious cycle in which patients lack limb strength and immobilization, postponing patients’ recovery from surgery. For this reason, early ambulation is considered one of the cornerstone components of enhanced recovery after surgery (ERAS) protocols [72], it`s recommend early mobilization to improve lung function and tissue oxygenation and avoid thromboembolic events [73]. Additionally, there is also proven evidence of benefits to the patients that enroll in prehabilitation intervention [74, 75]. Especially the multimodal therapy which has a combination of aerobic and resistance exercises, nutritional supplementation and psychological support [76].

Muscle fibers also influence the immunological response by controlling interleukin-6 and other peptides, regulating the synthesis of tumor necrosis factor-alpha and insulin resistance [77]. The reduction in skeletal muscle may cause immunosenescence, which is characterized by decreased cellular immunological function and increased inflammatory activity [78] in response to tumors, releasing pro-inflammatory cytokines and growth factors. A number of inflammatory indicators are reportedly prognostic factors of cancers, including the C-reactive protein-to-albumin ratio, neutrophil-to-lymphocytes ratio, and others [79, 80]. These inflammatory biomarkers are purportedly linked to the long-term survival of several cancer types, including esophageal neoplasms [81–84]. This inflammatory change might cause decreased host response to cancer [85] and may explain why sarcopenia impairs survival rates, as demonstrated in the current study’s findings.

Sarcopenia is also an indirect finding of the whole malnutrition status, comprising deficiency in the ingested amount of proteins, calories, minerals, and vitamins, all of which are essential for proper immune system function, cancer cells fighting, infections control, and healing processes [86, 87]. Hypoalbuminemia is one of the serum biomarkers of inadequate protein intake [88], and its relationship to unfavorable surgical results has been well established [89]. Albumin is involved in a range of physiological processes in the human body, including fluid kinetics and metabolism, and consequently, its deficiency is associated with numerous adverse postoperative outcomes [90]. Joliat et al. [91], in a recently published systematic review evaluating outcomes in gastrointestinal surgery, showed that low serum albumin was related to wound-related complications, acute respiratory distress syndrome, acute kidney injury, sepsis, anastomotic leak, ileus, and others.

In this sense, it is essential to discuss the available interventions for sarcopenia prevention, treatment, and decreasing its process before esophagectomy. Every esophageal cancer patient planning to undergo esophagectomy should be thoroughly evaluated for sarcopenia, where sarcopenia examination and severity classification should be purposefully undertaken to contemplate some prehabilitation strategies that aim to reverse the sarcopenia status before the surgery [92].

This study has some limitations. The definition of sarcopenia and the methodologies applied for measuring body composition employed in each study were heterogeneous, which is one of the study’s shortcomings. Several methods for evaluating sarcopenia have been proposed, such as lumbar skeletal muscle index, skeletal muscle mass index, psoas muscle index, low appendicular skeletal muscle mass index, and others. Besides, the cut point for differentiating sarcopenic and non-sarcopenic patients is still not well established. Most of the included studies used different cut points for women and men, considering the likely differences in muscle mass between these groups. The most frequently reported parameter and cutoff value used was lumbar skeletal muscle index (SMI) ≤ 38.5 cm^2^/m^2^ in women and ≤ 52.4 cm^2^/m^2^ in men. In a subgroup analysis, using only studies that applied this cut point for SMI, the statistical heterogeneity was reduced. However, other demographic variables aside from sex might also impact muscle mass, including ethnicity, age, and comorbidities, all contributing to clinical heterogeneity among the studies. Considering the presumed clinical heterogeneity, we used the random effect as the primary analysis model. However, sensitivity analysis with the fixed effect model in the subgroup analysis was consistent for most endpoints, demonstrating the robustness and validity of our findings, despite the study’s limitations.

## Conclusion

Sarcopenia is a highly significant preoperative comorbidity in patients submitted to esophagectomy for cancer. Preoperative sarcopenia imposes a higher risk for overall complications and severe complications. Besides, patients with sarcopenia had a lower chance of long-term survival.

### Electronic supplementary material

Below is the link to the electronic supplementary material.

Additional File 1: Subgroup analysis included only studies using Skeletal Muscle Mass Index (SMI) for assessing sarcopenia

Additional File 2: Newcastle-Ottawa Quality Assessment Form for Cohort Studies

## Data Availability

The datasets used and/or analysed during the current study available from the corresponding author on reasonable request.

## Abbreviations

SMM: Skeletal muscle mass
SMI: Skeletal muscle index
ICU: Intensive care unit
RD: Risk difference
MD: Mean differences
