# Supplementary material for: The impact of sarcopenia on esophagectomy for cancer: a systematic review and meta-analysis
Source: BMC Surg. 2023 Aug 17;23:240. doi: 10.1186/s12893-023-02149-6 (PMC10433615; doi:10.1186/s12893-023-02149-6)
Supplement: Supplementary file 1 — Additional File 1: Subgroup analysis included only studies using Skeletal Muscle Mass Index (SMI) for assessing sarcopenia [file 12893_2023_2149_MOESM1_ESM.docx]

|  |  | **Random effect model (Hedges)** | | | | **Fixed effect model (Inverse variance)** | | | |
| --- | --- | --- | --- | --- | --- | --- | --- | --- | --- |
|  |  |  | **95% CI** | |  |  | **95% CI** | |  |
| **Outcome** | **Number of studies** | **RD or MD** | **LL** | **UL** | **I^2^** | **RD or MD** | **LL** | **UL** | **I^2^** |
| **Postoperative mortality** | 9 | -0.02 | -0.10 | 0.06 | 92% | -0.00 | -0.03 | 0.02 | 0 |
| **Overall complications** | 11 | 0.07 | 0.01 | 0.14 | 36.5% | 0.07 | 0.02 | 0.13 | 42.5% |
| **Severe complications** | 5 | 0.09 | 0.02 | 0.17 | 29% | 0.09 | 0.03 | 0.15 | 18.5% |
| **Anastomotic leakage** | 9 | -0.02 | -0.04 | 0.01 | 0 | -0.02 | -0.04 | 0.01 | 49% |
| **Pneumonia** | 6 | 0.15 | 0.09 | 0.21 | 16.5% | 0.15 | 0.09 | 0.20 | 45.5% |
| **Hospital stay** | 6 | 0.12 | -0.04 | 0.28 | 0 | 0.12 | -0.04 | 0.28 | 0 |
| **Overall survival** | 10 | -0.18 | -0.31 | -0.06 | 78% | -0.18 | -0.21 | -0.10 | 64.5% |

**Supp. File 2.** Subgroup analysis included only studies using Skeletal Muscle Mass Index (SMI) for assessing sarcopenia (≤ 38.5 cm^2^/m^2^ in women and ≤ 52.4 cm^2^/m^2^ in men). RD: Risk difference; MD: Mean difference; CI: Confidence interval; LL: lower limit; UL: upper limit. Values highlighted in gray show significant differences.
