# Supplementary material for: The impact of sarcopenia on esophagectomy for cancer: a systematic review and meta-analysis
Source: BMC Surg. 2023 Aug 17;23:240. doi: 10.1186/s12893-023-02149-6 (PMC10433615; doi:10.1186/s12893-023-02149-6)
Supplement: Supplementary file 2 — Additional File 2: Newcastle-Ottawa Quality Assessment Form for Cohort Studies [file 12893_2023_2149_MOESM2_ESM.docx]

|  | | | **Selection** | | | **Comparability** | | | **Outcome** | | |
| --- | --- | --- | --- | --- | --- | --- | --- | --- | --- | --- | --- |
| **Author-Year** | **Representativeness of the exposed cohort** | **Selection of the non-exposed cohort** | | **Ascertainment of exposure** | **Demonstration that outcome of interest was not present at start of study** | **Comparability of cohorts on the basis of the design or analysis controlled for confounders** | **Assessment of outcome** | **Was follow-up long enough for outcomes to occur** | | **Adequacy of follow-up of cohorts** | **Score** |
| **NAMBARA M - 2021** | 1 | 1 | | 1 | 1 | 0 | 1 | 0 | | 0 | 5 |
| **FEHRENBACH U - 2021** | 1 | 1 | | 1 | 1 | 0 | 1 | 0 | | 0 | 5 |
| **UEMURA S - 2021** | 1 | 1 | | 1 | 1 | 0 | 1 | 1 | | 0 | 6 |
| **WAKEFILED CJ - 2021** | 1 | 1 | | 1 | 1 | 0 | 1 | 0 | | 0 | 5 |
| **MAYANAGI S - 2020** | 1 | 1 | | 1 | 1 | 0 | 1 | 0 | | 0 | 5 |
| **WANG PY - 2020** | 1 | 1 | | 1 | 1 | 0 | 1 | 0 | | 0 | 5 |
| **MAEDA N - 2020** | 1 | 1 | | 1 | 1 | 0 | 1 | 0 | | 0 | 5 |
| **SAKAI M - 2021** | 1 | 1 | | 1 | 1 | 0 | 1 | 1 | | 0 | 6 |
| **KAWAKITA Y - 2020** | 1 | 1 | | 1 | 1 | 0 | 1 | 0 | | 0 | 5 |
| **SRPCIC M - 2020** | 1 | 1 | | 1 | 1 | 0 | 1 | 0 | | 0 | 5 |
| **MENEZES TM - 2020** | 1 | 1 | | 1 | 1 | 0 | 1 | 0 | | 0 | 5 |
| **ISHIDA T - 2019** | 1 | 1 | | 1 | 1 | 0 | 1 | 0 | | 0 | 5 |
| **KAMITANI N - 2019** | 1 | 1 | | 1 | 1 | 0 | 1 | 0 | | 0 | 5 |
| **OGUMA J - 2019** | 1 | 1 | | 1 | 1 | 0 | 1 | 1 | | 0 | 6 |
| **XU J - 2019** | 1 | 1 | | 1 | 1 | 0 | 1 | 0 | | 0 | 5 |
| **YASSAIE SS - 2019** | 1 | 1 | | 1 | 1 | 0 | 1 | 0 | | 0 | 5 |
| **SOMA D - 2019** | 1 | 1 | | 1 | 1 | 0 | 1 | 0 | | 0 | 5 |
| **MATSUNAGA T - 2019** | 1 | 1 | | 1 | 1 | 0 | 1 | 0 | | 0 | 5 |
| **NAGATA K - 2018** | 1 | 1 | | 1 | 1 | 0 | 1 | 1 | | 0 | 6 |
| **NAKASHIMA Y - 2018** | 1 | 1 | | 1 | 1 | 0 | 1 | 1 | | 0 | 6 |
| **JARVINEN T - 2018** | 1 | 1 | | 1 | 1 | 0 | 1 | 0 | | 0 | 5 |
| **MAKIURA D - 2018** | 1 | 1 | | 1 | 1 | 0 | 1 | 0 | | 0 | 5 |
| **ELLIOTT JA - 2017** | 1 | 1 | | 1 | 1 | 0 | 1 | 0 | | 0 | 5 |
| **KUDOU K - 2017** | 1 | 1 | | 1 | 1 | 0 | 1 | 1 | | 0 | 6 |
| **PAIREDER M - 2017** | 1 | 1 | | 1 | 1 | 0 | 1 | 0 | | 0 | 5 |
| **GROTENHUIS BA - 2016** | 1 | 1 | | 1 | 1 | 0 | 1 | 0 | | 0 | 5 |
| **MAKIURA D - 2016** | 1 | 1 | | 1 | 1 | 0 | 1 | 0 | | 0 | 5 |
| **NISHIGORI T - 2016** | 1 | 1 | | 1 | 1 | 0 | 1 | 0 | | 0 | 5 |
| **TAMANDL D - 2016** | 1 | 1 | | 1 | 1 | 0 | 1 | 1 | | 0 | 6 |
| **HARADA K - 2016** | 1 | 1 | | 1 | 1 | 0 | 1 | 1 | | 0 | 6 |
| **IDA S - 2015** | 1 | 1 | | 1 | 1 | 0 | 1 | 0 | | 0 | 5 |
| **PANJE CM - 2019** | 1 | 1 | | 1 | 1 | 0 | 1 | 1 | | 0 | 6 |
| **SIEGAL SR - 2018** | 1 | 1 | | 1 | 1 | 0 | 1 | 0 | | 0 | 5 |
| **SAEKI H - 2018** | 1 | 1 | | 1 | 1 | 0 | 1 | 1 | | 0 | 6 |
| **MURNANE LC- 2021** | 1 | 1 | | 1 | 1 | 0 | 1 | 0 | | 0 | 5 |
| **COLCORD ME- 2021** | 1 | 1 | | 1 | 1 | 0 | 1 | 0 | | 0 | 5 |
| **KAMADA T - 2022** | 1 | 1 | | 1 | 1 | 0 | 1 | 1 | | 0 | 6 |
| **WATANABE A - 2022** | 1 | 1 | | 1 | 1 | 0 | 1 | 0 | | 0 | 5 |
| **KURITA D - 2022** | 1 | 1 | | 1 | 1 | 0 | 1 | 0 | | 0 | 5 |
| **SUGIMURA K - 2022** | 1 | 1 | | 1 | 1 | 0 | 1 | 0 | | 0 | 5 |

**Supplementary File 1.** Newcastle-Ottawa Quality Assessment Form for Cohort Studies.
